# A Cone Beam Computed Tomography (CBCT) evaluation of MB2 canals in endodontically treated permanent maxillary molars. A retrospective study in Indian population

**DOI:** 10.4317/jced.52716

**Published:** 2017-01-01

**Authors:** Heeresh Shetty, Subodh Sontakke, Freny Karjodkar, Pankaj Gupta, Ashish Mandwe, K.S Banga

**Affiliations:** 1 MDS, Assistant Professor, Department of Conservative Dentistry and Endodontics, Nair Hospital Dental College, Mumbai, India; 2 MDS, Associate Professor, Department of Dentistry Grant Medical College and Sir JJ Group of Hospitals, Mumbai, India; 3 MDS, Professor and Head, Department of Oral Medicine and Radiology, Nair Hospital Dental College, Mumbai, India; 4 MDS, Associate Professor, Department of Conservative Dentistry and Endodontics, Nair Hospital Dental College, Mumbai, India; 5 MDS, Professor and Head, Department of Conservative Dentistry and Endodontics, Nair Hospital Dental College, Mumbai, India

## Abstract

**Background:**

Current technological advances have allowed application of different study designs and techniques for investigation of dental anatomy. Some clinical studies have provided evidence that Cone Beam computed tomography (CBCT) scanning is an important resource in assessment of root canal systems notably to identify MB2 canals in maxillary molars as CBCT scans allow *in vivo* dental investigation in axial, sagittal and coronal planes simultaneously. The current study was undertaken to detect and evaluate filled/unfilled MB2 canals in endodontically treated, asymptomatic maxillary molars utilizing cone beam computed tomography (CBCT).

**Material and Methods:**

A retrospective study of 100 CBCTs of patients were underwent scanning for various treatment modalities, with asymptomatic endodontically treated permanent first and second maxillary molars were selected. History of root canal treatment varied from minimum of 1 year to a maximum of 10 years. Axial and paraxial images obtained were used to assess the presence of MB2 canal. Paraxial images were used to assess the periapical status.

**Results:**

Of the 100 scans, 66 were of permanent maxillary first molar and 34 were of permanent maxillary second molar. The incidence of MB2 canal was 86.36% in maxillary first molars and 29.4% in maxillary second molars. 77.19 % of maxillary first molars and 90% of maxillary second molars had an unfilled MB2 canal. 72.7% of maxillary first molars and 88.8% of maxillary second molars showed significant periapical radiolucencies in unfilled MB2 canals.

**Conclusions:**

MB2 canals were present in majority of cases and most of the unfilled MB2 canals showed evidence of periapical radiolucencies.

** Key words:**MB2 Canals, Cone Beam computed Tomography (CBCT), Filled /Unfilled canals, Endodontically treated teeth.

## Introduction

The complexity of root canal system is directly correlated with endodontic treatment and its outcome (1). Permanent maxillary first molars have one of the most complex root and canal anatomy. Existence of a second canal in the mesiobuccal (MB) root of maxillary molars has been a topic of numerous studies and its reported incidence is in the range of 33% and 96% (2,3). However, clinical detection of MB2 in maxillary molars (40%) is much lower than that of laboratory based reports (4,5). Although literature shows a high incidence of MB2 canal, Sempira, *et al.* (6) reported that only 33% of maxillary first molars had a negotiable MB2 canal, as determined by use of standard operating microscope *in vivo*. The most important causes of non-negotiable MB2 canal location are its narrowness, diffuse calcification, pulp stones debris and torturous pathways (7). It is generally accepted that a major cause of failure of root canal treatment is the inability to recognize presence of and to adequately treat all canals of the root canal system.

Current technological advances have allowed application of different study designs and techniques for investigation of dental anatomy. Some clinical studies have provided evidence that Cone Beam computed tomography (CBCT) scanning is an important resource in assessment of root canal systems notably to identify MB2 canals in maxillary molars (8), as CBCT scans allow *in vivo* dental investigation in axial, sagittal and coronal planes simultaneously (9).

Aim of this study was to evaluated filled/unfilled MB2 canals in endodontically treated, asymptomatic permanent maxillary molars using CBCT.

## Material and Methods

The present study protocol was approved by institutional ethical committee. A retrospective evaluation was conducted by examining CBCT scans of 100 patients who had reported to Nair Hospital Dental college, Mumbai, India to undergone scanning in the department of Oral and Maxillofacial Radiology for various treatment modalities especially pre/post evaluation of implant sites. Selection of scans was based on the case history and pre scan clinical evaluation.

The CBCT machine used was KODAK 9000 *(CMOS Sensor, continuous mode and,12-28 sec scan time,90-500 µm voxel size & 5x 3.5 cm FOV)*.

-Inclusion criteria

1) Patient’s age: 15 years and above.

2) Recorded history of endodontic treatment of minimum one year.

3) Asymptomatic endodontically treated tooth (i.e. absence of pain, absence of tenderness on percussion, absence of a draining sinus etc.)

Of the 100 CBCT scans which were selected, 66 were of maxillary first molars and 34 were of maxillary second molars.

All images were assessed independently by an endodontist and an oral radiologist. In case of non-agreement, a consensus was reached by discussion between the two.

Observers were instructed to examine the following;

1) Presence/absence of MB2 canals.

2) Number of filled /unfilled MB2 Canals.

3) Periapical condition in association with MB2 Canal.

4) Pattern of MB2 canal.

Calibration of observers was done by following above mentioned criterion with 5 random CBCT.

**Figure 1:**
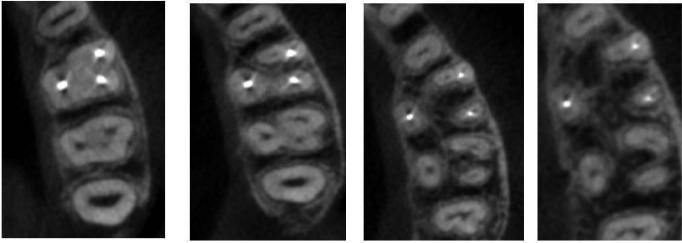
Successive slicing to detect the presence of MB2 canals.

Axial and paraxial images obtained were used to assess the presence of MB2 canal. Each mesiobuccal root image was scrolled axially from canal orifice to the radiographic apex. To be recorded as a second mesiobuccal canal, the canal had to be traceable in successive - sliced section to minimum half the length of the root (Fig. 1).

**Figure 2:**
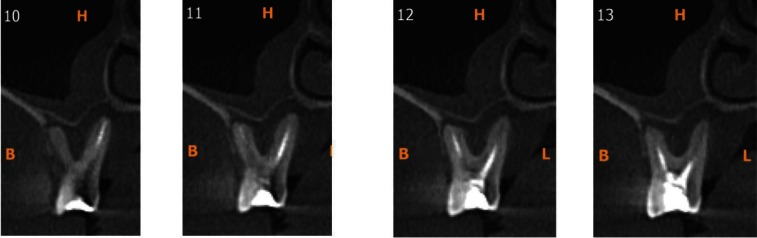
Para-axial section showing the periapical condition.

Paraxial images were used to assess the periapical status (Fig. 2). Periapical evaluation was done based on Cone Beam Computed Tomography Periapical Index Scores (CBCTPAI) given by Estrela *et al.* (10) in 2008. Scores of 0 and 1 were considered as negative (to negate healing lesions and PDL widening of healthy teeth examined by CBCT (11). Scores of 2 onwards were considered as positive.

**Figure 3:**
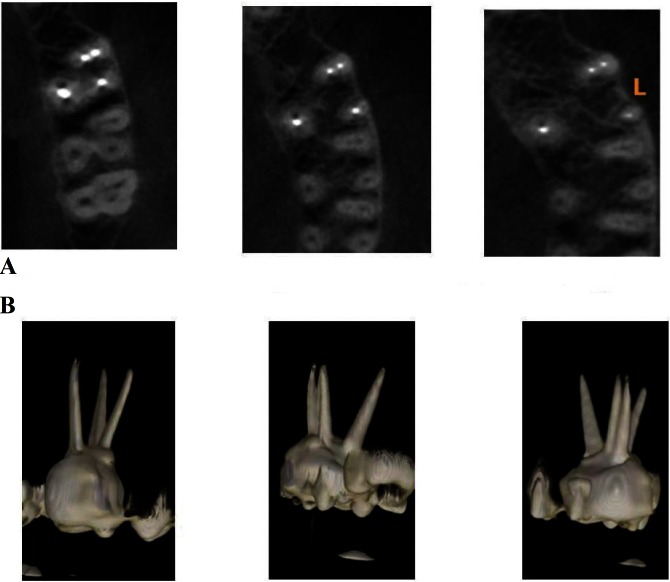
A) Consecutive slices showing an II pattern in a filled MB2 canal. B) 3D reconstructed images of the above teeth.

Pattern of the MB2 canals was categorized into two types, a) MB2 canals which merged with the MB1 canal which were represented by the “Y” b) MB2 canals which were distinct and had a separate opening at the apex were represented by “II” (Fig. 3a,b).

Any other finding of significance such as quality of obturation, periapical status of other roots which could influence the outcome of the endodontic treatment was also recorded.

Statistical analysis was performed using the Statistical Package for the social sciences version 20.0 (SPSS Inc., Chicago, II). Significance was set at *p* < 0.05.

## Results

In the present study all the 100 patients were asymptomatic for a period ranging from 1 to 10 years post endodontic treatment. 60 patients were asymptomatic for a period ranging from 1 to 4 years and 40 patients were asymptomatic for 4 to 10 years.

Of the 66 Maxillary first molars, MB2 canals were present in 86.36 %, absent in 12.1% and untraceable in 1.5%. Of the 57 cases where MB2 canals were present, 22.8% were filled and 77.19% were unfilled. Of the 13 canals filled, periapical radiolucency was present in 38.4% and absent in 61.5%. Of the 44 unfilled canals, periapical radiolucency was present in 72.7% and absent in 27.27 % (  Table 1). Of the 57 cases where MB2 canals were present, 71.9% showed Y pattern and 28.07 % showed II pattern ( Table 2).

**Table 1:**
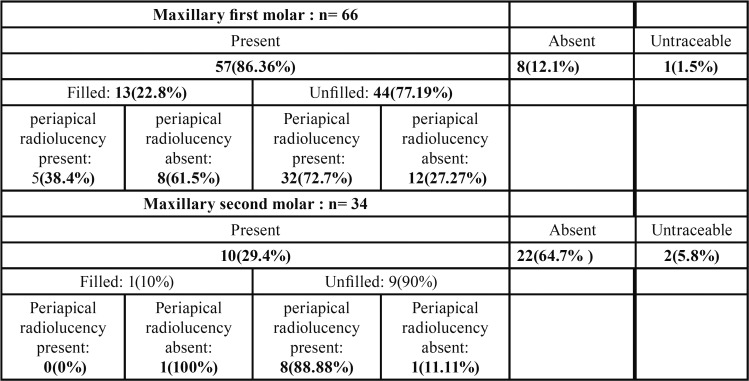
Observations of MB2 canals.

**Table 2:**
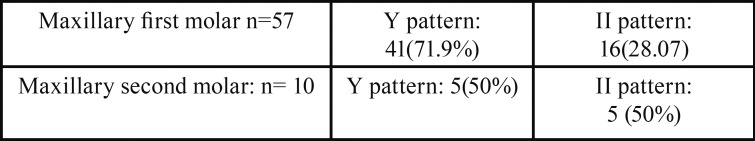
Observation of pattern of MB2 canals.

Of the 34 Maxillary second molars, MB2 Canals were present in 29.4%, absent in 64.7% and untraceable in 5.8%. Of the 10 cases where MB2 Canals were present, 10% were filled and 90% were unfilled. The 1 filled canal showed absence of periapical radiolucency. Of the 9 unfilled canals, periapical radiolucency was present in 88.8% and absent in 11.11%. ( Table 1). Of the 10 cases where MB2 Canals were present, both Y and II pattern were seen in equal percentage of cases ( Table 2).

Fisher’s exact test was performed to analyze relation of filled / unfilled canals with their periapical condition (presence / absence). In maxillary first molar, prevalence of periapical condition is significantly correlated with filled/unfilled condition. (Fisher’s exact test at 95% confidence interval; two-tailed, *P*=0.0442).

Prevalence of periapical condition and filled/unfilled condition in maxillary second molars was not correlated with each other (Fisher’s exact test at 95% confidence interval; two-tailed, *P*=0.200).

## Discussion

Comprehensive treatment of entire pulpal system dictates endodontic success. Variations in root and root canal morphology, especially in multi rooted teeth, are a constant challenge for diagnosis and management. Dentists need to have a thorough knowledge of root canal configurations and their variations for a successful endodontic treatment. A recent epidemiological study noted that 97% of 1,462,936 endodontically treated teeth were retained in symptom-free function over an 8-year follow-up period (12).

Mesiobuccal root of the maxillary molar contains a complex root canal system. *In vitro* studies have shown that a MB2 canal is present in more than 70% of maxillary first permanent molars (13,14). Histological evidence, however, suggests the presence of two MB canals approaching a remarkable 100 % (14). These systems communicate frequently along their lengths, and terminate separately in two or more apical foramina greater than 58 % of the time (15). It is hence hypothesized that the thorough clinician must, therefore, assume that all maxillary first molars have four canals until proven otherwise.

Given the limitations of conventional radiography for detection of apical periodontitis and availability of new emerging 3-dimensional imaging modalities, the Cone Beam Computed Tomography Periapical Index (CBCTPAI) was developed based on the criteria established by measuring periapical radiolucencies on CBCT scans. The CBCTPAI is a successor of Periapical Index (PAI) which was introduced by Ørstavik *et al.* (16) for the radiographic appraisal of endodontically treated teeth (11).

Healing of apical periodontitis is a dynamic process, and sufficient time is required to evaluate its progression and completion. Observations after a short follow-up may demonstrate only signs of healing. Therefore, results of studies with short follow-up periods may be skewed and not reflect the true prognosis (17). Follow-up of at least 1 year is required to reveal meaningful changes (18,19) but extension of follow-up to 3 or 4 years may be required to record a stable treatment outcome (20).

Weine’s classification has been used to describe four common configurations of the maxillary MB root. Type I is a single canal from orifice to apex, Type II has two orifices that converge to one, Type III has separate and distinct canals from orifice to apex, and Type IV begins as one canal and diverges into two separate canals.

The CBCT PAI System was used to assess their periapical condition. Weine’s Type II and Type III classification was used to determine the presence of MB2 canals and their pattern as both these types had two orifices in the mesiobuccal root. Weine’s Type IV was considered as a variation of the primary mesiobuccal canal in this study.

I. Prevalence of MB2 canals: The presence of MB2 canal was detected in 86% in maxillary first molars and 29% in maxillary second molars. These results are compared to the various MB2 assessment studies using CBCT (T Table 3).

**Table 3:**
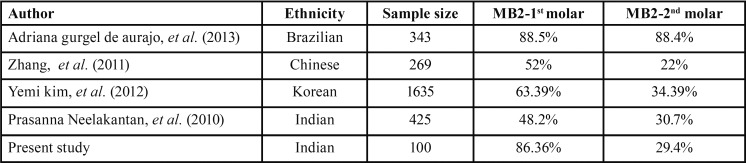
The various MB2 assessment studies using CBCT.

Though the results of this study falls within the wide range for presence of MB2 canals (33%-96%) in maxillary molars there is a wide contrast to recent CBCT studies for the same. This may be due to 1) Difference in CBCT equipment and parameters 2) Radiographic interpretation criteria 3) Ethnicity 4) Present study was conducted on endodontically treated tooth whereas previous studies were conducted on untreated teeth and hence the presence of the obturating material may hinder detection.

II. Filled/unfilled canals: Of the MB2 canals which were present only 22% were filled in first molars and 10% in second molar which correlates with the findings by Sempira and Hartwell (6) and other studies(4,5) that less than 40% of the MB2 canals are detected/negotiated clinically. However, results of the present study were lower than that of Burhley *et al.* (21) where the findings for the microscope, dental loupes and no magnification groups was 71.1%, 62.5% and 17.2%, respectively. Baldassari-Cruz *et al.* (22) reported that the percentage of second mesiobuccal canals was 82% under magnification. This lower incidence in this study may be due to 1) Canal to be recorded as an MB2 had to be traced in successive sliced section to minimum half the length of the root. 2) only 4% of endodontic treatment in the study was done with any kind of magnification.

III. Relation of the filled/Unfilled canals with their periapical condition: Of the treated MB2 canals in maxillary molars 62% showed absence of periapical radiolucencies and 38 % showed presence of periapical radiolucencies. The relatively high incidence of periapical radiolucencies may be due to 1) large number of these cases had inadequate obturation of the MB1/MB2 canals. 2) Periapical pathosis was still healing or had healed with periapical scar. 3) Other factors like resistant bacteria in the MB2 canal following treatment or new bacteria entering the root canal system via caries, cracks or restorative breakdown. The unfilled canals showed periapical radiolucencies in 73% cases. The above values were statistically significant (*p*= 0.0442). In second molars 89% of unfilled canals showed periapical radiolucencies. The only filled canal showed absence of periapical radiolucency. The above values were statistically insignificant (*p*= 0.2) mainly due to low sample size.

IV. Pattern of MB2 canal: In the MB roots of the maxillary first molar with additional canals 72% joined the main canal (Type II) which is in agreement with the various Chinese, Caucasian, Korean and Brazilian studies (23-28). Unlike the canal configuration in the first molars, 50% MB Roots of second molars showed two separate apical foramen (Type III). The results of this study are comparatively high when compared to 24.4% recorded for Type III in a study conducted in an Indian population (8) may be primarily due to the smaller sample size of the maxillary second molars in this study.

## Conclusions

Within the limitations of this study it can be concluded that the majority of the MB2 Canal present were unfilled in endodontically treated maxillary molars in an Indian population. Most of these unfilled MB2 canals showed evidence of periapical radiolucencies.
